# Systems Biology Approaches Toward Understanding Primary Mitochondrial Diseases

**DOI:** 10.3389/fgene.2019.00019

**Published:** 2019-02-01

**Authors:** Elaina M. Maldonado, Fatma Taha, Joyeeta Rahman, Shamima Rahman

**Affiliations:** ^1^Mitochondrial Research Group, UCL Great Ormond Street Institute of Child Health, London, United Kingdom; ^2^Metabolic Unit, Great Ormond Street Hospital for Children NHS Foundation Trust, London, United Kingdom

**Keywords:** integrative omics, genome scale models, constraint based modelling, network biology, mitochondrial disease, diagnostics, biomarkers, novel therapy development

## Abstract

Primary mitochondrial diseases form one of the most common and severe groups of genetic disease, with a birth prevalence of at least 1 in 5000. These disorders are multi-genic and multi-phenotypic (even within the same gene defect) and span the entire age range from prenatal to late adult onset. Mitochondrial disease typically affects one or multiple high-energy demanding organs, and is frequently fatal in early life. Unfortunately, to date there are no known curative therapies, mostly owing to the rarity and heterogeneity of individual mitochondrial diseases, leading to diagnostic odysseys and difficulties in clinical trial design. This review aims to discuss recent advances and challenges of systems approaches for the study of primary mitochondrial diseases. Although there has been an explosion in the generation of omics data, few studies have progressed toward the integration of multiple levels of omics. It is evident that the integration of different types of data to create a more complete representation of biology remains challenging, perhaps due to the scarcity of available integrative tools and the complexity inherent in their use. In addition, “bottom-up” systems approaches have been adopted for use in the iterative cycle of systems biology: from data generation to model prediction and validation. Primary mitochondrial diseases, owing to their complex nature, will most likely benefit from a multidisciplinary approach encompassing clinical, molecular and computational studies integrated together by systems biology to elucidate underlying pathomechanisms for better diagnostics and therapeutic discovery. Just as next generation sequencing has rapidly increased diagnostic rates from approximately 5% up to 60% over two decades, more recent advancing technologies are encouraging; the generation of multi-omics, the integration of multiple types of data, and the ability to predict perturbations will, ultimately, be translated into improved patient care.

## Introduction

Mitochondria are double membraned sub-cellular structures that perform multiple cellular and metabolic functions including the production of reducing equivalents through the tricarboxylic acid (TCA) cycle and fatty acid β-oxidation, maintenance of calcium homeostasis, nutrient signalling through mTOR, AMPK and other pathways, and the production of reactive oxygen and nitrogen species (Rahman and Rahman, 2018). The best studied and most well-known function of mitochondria, however, is the production of energy in the form of ATP through the process of oxidative phosphorylation (OXPHOS). The pumping of protons through four enzyme complexes (complexes I–IV) results in an electrochemical gradient across the inner mitochondrial membrane which in turn generates a chemiosmotic force that is utilised by the fifth complex, the F_1_F_0_-ATP-synthase, to phosphorylate ADP to ATP (Hatefi, 1985). OXPHOS requires the coordinated effort of greater than 400 proteins (Pagliarini et al., 2008) including the subunits and assembly factors of the five multi-subunit enzyme complexes, mobile electron carriers, and protein and nucleotide transporters. Whilst most OXPHOS proteins and the majority of the ∼1500 mitochondrial proteins (Calvo et al., 2016) are encoded in the nuclear genome, mitochondria also have endogenous mitochondrial DNA (mtDNA) as a result of an endosymbiotic event nearly 2 billion years ago (Gray et al., 1999). The mitochondrial genome is a small circular genome present in multiple copies, encoding 13 protein-coding genes, all of which are OXPHOS subunits, as well as 24 tRNA and rRNA genes to enable their translation (Anderson et al., 1981).

Both genomic and mtDNA mutations can lead to primary mitochondrial disorders which represent one of the most common (prevalence of 1:5000) and debilitating inherited metabolic diseases (Gorman et al., 2015), often resulting in early mortality. To date, more than 350 genes have been causally linked to mitochondrial disease (Rahman and Rahman, 2018). The genetic landscape of mitochondrial disorders is not only complicated by the involvement of multiple genomes, but also by the co-existence of mutant and wild-type mtDNA molecules in different cells and tissues, a phenomenon known as heteroplasmy (Stewart and Chinnery, 2015). Furthermore, patients often present with multi-systemic disease, especially affecting organs with high bioenergetic demands such as the brain (Rahman, 2015; Pitceathly, 2016; Hikmat et al., 2017), heart (Götz et al., 2011; Enns, 2017), muscle (DiMauro et al., 1985; Holt et al., 1988; Pfeffer and Chinnery, 2013) and liver (McKiernan et al., 2016). A number of mitochondrial gene defects can be associated with defined clinical syndromes, such as mitochondrial encephalopathy lactic acidosis and stroke-like episodes (MELAS), which is usually associated with a specific mutation in the mitochondrial leucine tRNA (El-Hattab et al., 2015), or Leigh syndrome, an encephalomyelopathy characterised by bilateral basal ganglia lesions, which can be caused by defects of more than 89 different mitochondrial and nuclear genes (Leigh, 1951; Rahman et al., 2017). Most mitochondrial diseases, however, do not fit into a classical syndromic presentation and can affect nearly any organ or system in the body in any combination (Munnich et al., 1992). Moreover, mutations in the same gene can lead to contrasting clinical presentations between patients, even within a single family. While OXPHOS dysfunction certainly contributes to disease pathophysiology, especially in patients with isolated or combined OXPHOS complex deficiencies, it is evident that a multitude of impaired mitochondrial functions contribute to disease. These include imbalanced mitochondrial dynamics (Janer et al., 2016), aberrant mitochondrial lipid homeostasis (Wortmann et al., 2012), deficiencies of vitamin and cofactor metabolism (Duncan et al., 2009), and altered redox ratios (Khan et al., 2014; Titov et al., 2016). Many aspects of mitochondrial dysfunction also contribute to the pathophysiology of cancer (Warburg et al., 1927; Vyas et al., 2016), neurodegenerative disorders (Lin and Beal, 2006; Grunewald et al., 2018), and organismal ageing (Bratic and Larsson, 2013). The genetic, pathophysiological, and clinical heterogeneity observed in mitochondrial disorders have resulted in diagnostic odysseys and a lack of curative therapies for affected patients, contributing to an overall poor prognosis.

**FIGURE 1:**
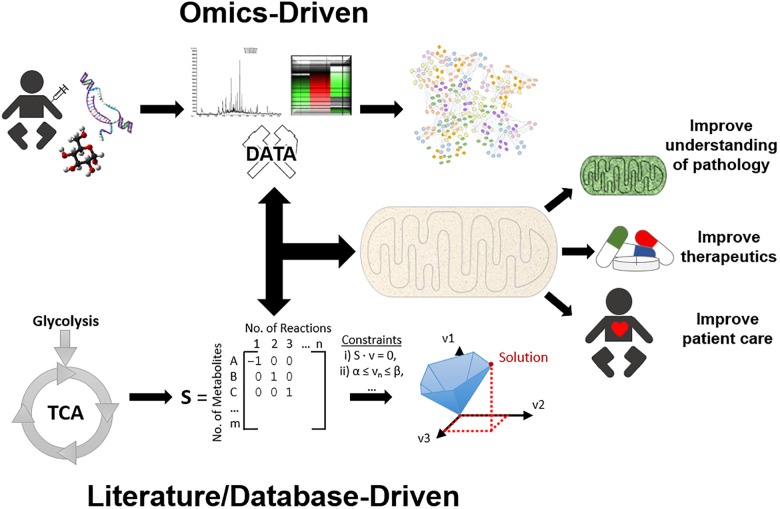
An overview of systems approaches applied for mitochondrial research. To simplify systems-based approaches, they can be categorised into two main approaches, top-down and bottom-up. The top-down workflow can simplistically be described as samples that have been collected, processed by high throughput methods, and analysed by bioinformatics, e.g., protein network analysis, to gain a better understanding of function. On the other side of the spectrum, the bottom-up workflow can be described as identifying molecular data, formatting this information into a genome scale metabolic model (GEM), and utilising constraint-based modelling (CBM) to predict solutions and gain a better understanding of mechanisms. However, in practice the researcher must use whatever data is sufficiently available at any level of organisation, and build up/down/across to other levels, known as middle-out. Together, these systems approaches can aid in mitochondrial research by providing further insight into mitochondrial diseases, therapeutic approaches, and ultimately improving patient health care.

An improvement in diagnostic and therapeutic outcomes requires an enhanced understanding of mitochondrial function and pathophysiology. In recent years, systems biology, or the use of computational and mathematical methods to model complex biological systems, has emerged as a valuable tool to analyse and characterise complex cellular and organellar relationships in health and disease states. Biological networks can be created using experimental “omics” datasets (e.g., genomics, transcriptomics, proteomics, metabolomics, and epigenomics) (Topol, 2014) as scaffolds to construct models (Baumgart et al., 2016; Lienhard et al., 2017; Ali et al., 2018). This approach enables identification of novel pathomechanisms, but can also be used to identify novel biomarkers and therapeutic targets for mitochondrial disease (Suomalainen et al., 2011). Conversely, the modelling of biological systems can be achieved by constructing GEnome-scale Metabolic models (GEMs) from pre-existing database and literature inputs rather than using novel experimental data (O’Brien et al., 2015; Brunk et al., 2018). The latter approach aims to fully encompass all interactions within a system. These models can then be subjected to manipulations and provide another means for predictive modelling. In the context of mitochondrial disorders, this approach can be exploited to observe the functional consequences of aberrant genetic changes or to identify novel therapeutic points of intervention which could facilitate targeted novel drug design or orphan drug repurposing. Additionally, the combination of top-down and bottom-up approaches have proved to be powerful tools to effectively integrate experimental data with the compendium of literature to gain a holistic understanding of a complex biological system (Sun et al., 2018). In practice, model reconstructions begin from whichever level is most rich with data, and build up or down to other levels as required (Noble, 2002), see Figure 1. Network-based approaches therefore are powerful tools to study mitochondrial function (and dysfunction) as they facilitate the visualisation and manipulation of multitudinous interactions between genes, transcripts, proteins, and metabolites. This enables the elucidation of integrative mitochondrial functions and can expedite the discovery of novel interactions which otherwise may have been missed using traditional experimental techniques. These approaches will ultimately have beneficial implications for developing novel diagnostic and therapeutic strategies for mitochondrial disease, refer to Box 1 for key terms. This review will discuss the recent advances that systems approaches have contributed to an overall understanding of mitochondrial biology and pathophysiology, as well as the limitations of these approaches and some of the remaining challenges in the field.

**BOX 1. Glossary of key terms.**

Biological network: a graphical representation of interacting moieties typically depicted as nodes (circles) and edges (lines).

Constraint-based modelling (CBM): a modelling approach that computes mathematical relationships imposed by a set of constraints.

Flux balance analysis (FBA): a constraint-based modelling method that uses linear programming to maximise the objective function, and computes the set of fluxes through the network while satisfying all defined constraints.

Flux variability analysis (FVA): a constraint-based modelling method that computes the minimum and maximum range of each reaction flux through a metabolic network while satisfying all defined constraints.

GEnome-scale Metabolic model (GEM): a mathematical, structured network of chemical reactions to represent the metabolism of a living organism based on its genome and the literature. A GEM can be structured based on defined model constraints which, if known, can include their stoichiometry, thermodynamics, enzymatic capacity, localisation, and functional annotation of gene-protein-reaction association, and other known constraints.

Interactome: a set of all known interactions involved in a particular system studied.

Mitochondrial protein functional (MPF) network: a network modality generated considering protein functions and their subsequent network position.

Network position: a numerical score for nodes within a network ranging from 0 to 1 in order of most central (0) to most peripheral (1) node, corresponding to contribution of that node to network functionality.

Node: an interacting molecule (gene, protein, etc.) in a biological network.

Objective function: a function (or a target reaction/flux) that is desired to be maximised or minimised.

Protein–protein interaction network (PPIN): common network representation in systems biology of all known proteins within a particular system, and all their interactions.

Solution space: a set of all possible values (or solutions) of an optimisation problem that satisfy the problem’s constraints.

Systems biology: an interdisciplinary science which uses mathematical and computational methods, by *in silico* simulations, to aid the understanding of complex biology by elucidating emergent properties once the system is studied as a whole, rather than in parts.

Transcriptome-metabolome-wide association study (TMWAS): an association study between two layers of omics data, transcriptomic and metabolomic data.

## Advances in Systems Mitochondrial Biology

The advent of multi-omics techniques in mitochondrial biology has given rise to a vast amount of large, complex datasets (Rahman and Rahman, 2018). A large number of these datasets can be found online, including UniProt (The UniProt, 2017), Kyoto Encyclopedia of Genes and Genomes (Kanehisa et al., 2016), the Human Protein Atlas (Thul et al., 2017), and mitochondrial databases including MitoCarta (Calvo et al., 2016) and MitoMiner (Smith and Robinson, 2016), in addition to many other mitochondrion-specific databases as listed in Table 1. However, the enormity of these data creates considerable challenges in drawing meaningful conclusions. The development of sophisticated bioinformatics pipelines has enabled the management and analysis of large complex datasets and facilitated meaningful biological interpretation (Luscombe et al., 2001). Although bioinformatics can provide additional insight by re-analysing experimental data, it is limited in its ability to predict behaviour of complex systems. More recently, predictive computational biology has become a fundamental part of systems approaches. It provides a natural continuation within experimental biology to elucidate complex, synergistic, interactive behaviours that underpin emergent properties from a biological system studied as a whole (Kitano, 2002; Pagliarini and Rutter, 2013). For application purposes, several complementary tools can be used in systems approaches, e.g., multiple dataset analyses, omics integration tools, GEMs and constraint-based modelling (CBM), to incorporate and reconcile the increasingly available independent, diverse datasets.

**Table 1:** Human Mitochondrial Databases.

Database	Content	Website	Last update
HmtDB	Human mitochondrial genome sequences annotated with population and variability data	www.hmtdb.uniba.it	2018
Integrated Mitochondrial Protein Index (IMPI) Q2 2018	A collection of genes that encode proteins with strong evidence for cellular localisation within the mammalian mitochondrion.	http://www.mrc-mbu.cam.ac.uk/impi	2018
MitoMap	Polymorphisms and mutations in human mDNA	www.mitomap.org	2018
MitoMiner 4.0	Mitochondrial localisation evidence and phenotype data for mammals, zebrafish and yeasts	mitominer.mrc-mbu.cam.ac.uk	2018
Human MitoCarta 2.0	Inventory of nuclear and mtDNA genes encoding proteins with strong support of mitochondrial localisation	https://www.broadinstitute.org/files/shared/metabolism/mitocarta/human.mitocarta2.0.html	2017
MitoBreak	mDNA breakpoints	http://mitobreak.portugene.com/cgi-bin/Mitobreak_home.cgi	2017
MitoDB	Information regarding the clinical features seen in mitochondrial diseases.	mitodb.com	2016
MitoAge	Calculated mtDNA compositional features of the entire mitochondrial genome, mtDNA coding and non-coding regions, codon usage for each protein-coding gene, and longevity records for over 900 species from all taxa of the Kingdom Animalia.	http://www.mitoage.info/	2016
MitoProteome	An object-relational mitochondrial gene/protein sequence database and annotation system	www.mitoproteome.org	2016
The EMPOP database	The collection, quality control and searchable presentation of mtDNA haplotypes from all over the world	https://empop.online/	2015
MitoGenesisDB	Mitochondrial spatio-temporal expression through global mRNA analyses	http://www.dsimb.inserm.fr/dsimb_tools/mitgene/biologicalbackground.php	2010
Mitochondrial tRNA database - tRNAdb	Mitochondrial tRNA genes	http://mttrna.bioinf.uni-leipzig.de/mtDataOutput/	2009
Human Mitochondrial Protein Database (HMPDb)	Mitochondrial and human nuclear encoded proteins involved in mitochondrial biogenesis and function.	https://bioinfo.nist.gov/	2007
Mamit-tRNA	Mammalian mitochondrial tRNA genes	http://mamit-trna.u-strasbg.fr/	2007
mtDB	Complete mitochondrial genomes since early 2000	www.mtdb.igp.uu.se	2007
GiiB-JST mtSNP	Information related to the functional differences among mitochondrial SNPs	http://mtsnp.tmig.or.jp/mtsnp/index_e.shtml	2006


*A list of databases in English that relate to human mitochondria research, which still appear to be active*.

### Multi-Omics Approaches to Understanding Mitochondrial Function

#### Omics as a Tool in Mitochondrial Research

Systems biology can be used to answer several outstanding (patho)physiological mitochondrial questions including the basic biology of mitochondrial pathways, the pathophysiology underpinning primary mitochondrial disease; and also in models of neoplasms and ageing. Utilising global non-biassed datasets acquired from high-throughput assays or “omics” datasets is one method of enhancing our understanding of complex biological systems. The majority of these types of studies have focussed linearly on one type of analysis and make inferences [reviewed in (Rahman and Rahman, 2018)]. Significant advances have been made in revealing novel aspects of mitochondrial structure and function such as the mitochondrial phosphorylation targets of AMPK (Toyama et al., 2016) or the elucidation of the step-wise assembly of eukaryotic mitochondrial complex I (Stroud et al., 2016). Furthermore, omics-based analyses have been instrumental in understanding disease mechanisms beyond OXPHOS dysfunction/energy deficit which often is insufficient in explaining the clinical phenotypes observed in patients (Thompson Legault et al., 2015; Esterhuizen et al., 2018; Rahman and Rahman, 2018). A notable example is the alteration of one-carbon metabolism in mtDNA maintenance defects in mouse of models of adult and paediatric mitochondrial disease (Nikkanen et al., 2016; Khan et al., 2017).

#### Integrative Genomics

More than a decade ago, the availability of genome-scale data from *Saccharomyces cerevisiae* enabled yeast biologists to begin to integrate large-scale functional genomics data to identify candidate mitochondrial disease genes (Steinmetz et al., 2002; Prokisch et al., 2004; Aiyar et al., 2008). At around the same time, the Mootha group at Harvard integrated data from diverse genome-scale data sets using a Bayesian mathematical model, to predict the probability of a mitochondrial function for a specific candidate protein (Calvo et al., 2006). The resulting compendium, named ‘Maestro’, used 8 data sets to compute the likelihood of mitochondrial localisation of 33860 proteins listed in the Ensembl human genome database: presence of an N-terminal mitochondrial import sequence, as predicted by the TargetP programme; presence of protein domains suggesting mitochondrial function, as predicted by the MitoPred programme; *cis*-regulatory motifs containing binding sites for any of 3 transcription factors (Errα, Gapba, and NRF1) located within 2 kb upstream of the gene; homology to one or more of 749 mitochondrial proteins encoded by the *S. cerevisiae* genome; ancestral bacterial homology to proteins in *Rickettsia prowazekii*, thought to be the closest bacterial progenitor of mitochondria; co-expression with genes known to encode mitochondrial proteins; tandem mass spectrometry survey of the mouse mitochondrial proteome; transcriptional activation during a cellular model of mitochondrial biogenesis, in which mitochondrial proliferation was stimulated by exposure to the mitochondrial transcriptional co-activator PGC1-α. Maestro correctly predicted 71% of known mitochondrial proteins when it was first devised. More recent iterations of this compendium, known as MitoCarta and MitoCarta 2.0 (Pagliarini et al., 2008; Calvo et al., 2016), are even more comprehensive and have underpinned the mitochondrial genomic diagnostic revolution (Rahman and Rahman, 2018). It is anticipated that new data and techniques will facilitate development of a complete mitochondrial proteome catalogue in the fullness of time, including tissue-specific databases that might explain tissue-specific manifestations of particular mitochondrial gene defects.

#### Combining Multi-Omic Datasets

Multi-omics studies are emerging as valuable tools to gain a dynamic understanding of the mitochondrion. Integrative omics approaches as opposed to single-omics based techniques are beneficial as they increase the certainty of a biological finding if it can be validated by concordant multiple omics signatures (genomic, transcriptomic, and proteomic). Frequently, transcriptomic and proteomic signatures do not correlate; thus making endpoint phenotypic inferences based on untargeted transcriptome analyses with no prior associations can be inaccurate or misleading (Wu et al., 2014). This can partially be explained by the fact that high-throughput discovery techniques largely focus on endpoint measurements (transcript/protein level) and cannot accurately account for transcriptional and translational control events which may alter protein expression and downstream functional consequences (Fisher-Wellman et al., 2018). Meaningful functional interpretations can be aided with specialist assays which can be compatible with omics techniques to observe real-time phenotypic changes in response to global transcriptomic/proteomic remodelling. Genome-wide CRISPR-Cas9 screening is one such technique which has been used in conjunction with genomics to identify novel genes involved in OXPHOS (Arroyo et al., 2016). Multi-omics studies can also be aided by functional phenotyping techniques which are compatible with high-throughput screens such as a novel assay suite developed to measure mitochondrial bioenergetics parameters in real-time (Fisher-Wellman et al., 2018).

Multi-layered omics in recent years has been instrumental in elucidating the molecular basis of complex mitochondrial processes including the activation of the mitochondrial unfolded protein response (Wu et al., 2014), identifying transcription factor ATF4 as a regulator of mitonuclear stress (Quirós et al., 2017), the involvement of mitoribosomes and complex IV subunits in T-cell activation (Tan et al., 2017) and the role of mitoprotease Oct1p as a novel regulator of Coenzyme Q_10_ biosynthesis (Veling et al., 2017). Integrated omics are useful in mitochondrial physiology to discriminate between primary disease-causing mechanisms and compensatory mechanisms. This was recently demonstrated by a study conducted by Mootha and colleagues wherein multi-omic profiling of benign mitochondria-rich renal tumours showed a selective loss-of-function of complex I which in turn led to compensatory glutathione biosynthesis (Gopal et al., 2018). At present, these integrated studies have been limited to cell and animal models (Wu et al., 2014; Kühl et al., 2017; Quirós et al., 2017; Tan et al., 2017; Veling et al., 2017; Lapointe et al., 2018; Lee et al., 2018). However, this body of work highlights the power of systems approaches and integrative omics in elucidating novel mechanisms and will undoubtedly be invaluable in studies of mitochondrial disease pathology in relevant patient cohorts and animal disease models as they become increasingly available.

#### Interactome Models

Another approach, which has become central to systems biology, has been to build ‘interactome’ networks based on known protein–protein interactions. Early examples of these protein-protein interaction networks (PPINs) include ‘MitoInteractome’ and InterMitoBase (Reja et al., 2009; Gu et al., 2011). MitoInteractome used homology-based interaction modelling across 74 species to produce a database of 6,549 protein sequences (Reja et al., 2009), whilst InterMitoBase mined a range of resources to create a compendium of 5,883 protein–protein interactions between 2,813 proteins (Gu et al., 2011). Another interactome model, the Mitochondrial Protein Functional (MPF) network, built on the observation that the spatial organisation of mitochondrial proteins is linked to function, and localised mitochondrial proteins known to interact with other mitochondrial proteins at the centre of the network, and mitochondrial proteins interacting with non-mitochondrial proteins at the periphery of the network (Yang et al., 2013). The MPF network aimed to use network position (scored 0–1, with more centrally localised proteins scoring closer to 0 and peripheral proteins closer to 1) to represent submitochondrial localisation of 1,254 mitochondrial proteins (Yang et al., 2013). The MPF was validated by finding proteins with core mitochondrial functions such as OXPHOS and fatty acid β-oxidation at the centre of the network (e.g., the short/branched chain acylCoA dehydrogenase ACADSB had a score of 0.062), whereas proteins linked to mitochondrial biogenesis and apoptosis were peripherally located (e.g., the mitochondrial fission factor MFF scored 0.935). Furthermore, network position was highly correlated with mitochondrial compartment – matrix and inner mitochondrial membrane proteins had central network positions, whereas outer mitochondrial membrane proteins were peripherally located in the network. There also appeared to be correlations between disease genes within the network. For example, five genes associated with MELAS had a network position of 0.01, five genes causing pyruvate dehydrogenase (PDH) deficiency had an average score of 0.03 (±0.01), and 21 genes associated with Leigh syndrome had an average network position of 0.10 (±0.09). MELAS, PDH deficiency and Leigh syndrome are all considered primary mitochondrial disorders. In contrast Charcot-Marie-Tooth disease type 2, caused by a defect of axonal mitochondrial transport, had a more peripheral network position with a score of 0.68 (Yang et al., 2013). The authors then went on to use the MPF network to try to predict candidate mitochondrial disease genes, but this approach will need to be finessed as the MPF becomes more sophisticated by the addition of further data. Another application of interactome modelling was the identification of drug targets that could rescue a cellular model of Parkinson’s disease caused by the MPP^+^ toxin, an inhibitor of mitochondrial complex I (Keane et al., 2015).

#### Other Network Approaches

Molecular networks can also be used to derive gene ontologies, and this approach was employed to develop the Ingenuity Pathway Analysis software, that uses algorithms to infer omics networks based on functional similarity (Calvano et al., 2005). Disease-specific networks (‘diseasomes’) are increasingly being developed, including for many cancer types, orphan diseases and inborn errors of metabolism (Goh et al., 2007; Barabasi et al., 2011; Zhang et al., 2011). Organ-specific networks are also being created, such as The Virtual Brain, which simulates primate brain network dynamics and holds the promise of a neuroinformatics-based personalised medicine strategy for neurological disorders (Sanz Leon et al., 2013; Falcon et al., 2016). So far, there do not appear to be any mitochondrial disease specific networks, but this is likely to change as multi-omics data sets are generated from larger patient cohorts affected by primary mitochondrial diseases (Rahman and Rahman, 2018). Another emerging field is that of network pharmacology, where molecular networks are being used to screen drugs for efficacy *in silico* before wet lab testing begins, to try to reduce the costs associated with drug development (Guney et al., 2016).

The aim of network biology is to provide an ‘eagle eye’ view of the system using *in silico* simulations, and can be applied to view multi-omic datasets. The field of biological networks is expanding exponentially and, going forward, the integration of multiple omics data sets, including genomic, transcriptomic, proteomic, metabolomic and phenomic data, will increase the power of network biology to identify disease mechanisms, biomarkers, and novel treatments (Stevens et al., 2014; Rahman and Rahman, 2018). Previously few tools were able to integrate more than two omics data sets. This is because the different topological features of different omics datasets may render them unable to identify community structures within networks and observe network changes in response to perturbations (e.g., treated versus untreated, or healthy versus diseased) (Uppal et al., 2018). In addition, the integrative analysis of large omics datasets may lead to fitting problems (Liang and Kelemen, 2017). Newer methods such as the Similarity Network Fusion (SNF) are able to aggregate and analyse multiple data sets on a genomic scale (Wang et al., 2014). The fused similarity network is composed of nodes (patients) positioned based on similarity; the greater the similarities between two nodes (a function of all inputted data), the closer they are positioned. The resulting network of clusters of similar patients can then be used to derive information about the very basic molecules that caused these patient clusters to form. If these molecules are consistently present in more than one patient, the SNF can be inferred to be highlighting pertinent disease-related molecules (Wang et al., 2014). In addition, several frameworks are emerging to accommodate the integration of multiple complex omics datasets to visualise data and evaluate changes under different physiological conditions and these have been successfully applied in cancer and immunology (Mo et al., 2013; Argelaguet et al., 2018; Bakker et al., 2018; Forsberg et al., 2018). Recently the xMWAS software has been developed as a new tool to integrate, visualise and analyse up to four omics datasets, by using a partial least squares regression algorithm (Uppal et al., 2018). One application of xMWAS was to analyse mitochondrial mediated toxicity to the toxins paraquat and maneb, and to demonstrate that these exert their mitochondrial toxicities by different molecular mechanisms, as elucidated by global remodelling of the nuclear transcriptome and metabolome. Clustering analysis revealed that paraquat toxicity induced increased antioxidant production, stress response, and mitochondrial biogenesis (Go et al., 2018). Although the molecular mechanisms of paraquat toxicity have been well-characterised (Tawara et al., 1996), the reliable *in silico* reproducibility of complex pharmacological phenomena can hopefully be used in future to supplement multi-omics mitochondrial studies to identify novel biomarkers and therapeutic targets.

### Genome-Scale Metabolic Models

**FIGURE 2:**
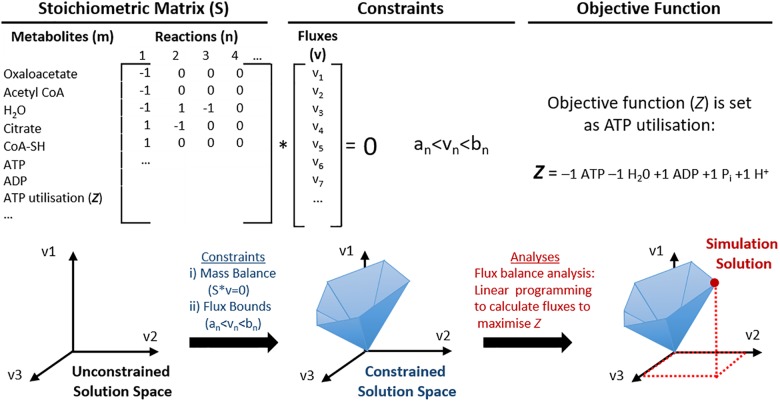
Schematic representation of the utilisation of genome scale metabolic models (GEMs) for constraint-based modelling (CBM). Mitochondrial metabolic pathways can be represented as a list of stoichiometric formulas and converted into a large stoichiometric matrix (S). In this format, the model does not have any constraints, thus the solution space may even include biologically irrelevant solutions. Constraints are then applied to utilise constraint-based modelling (CBM); such as (i) mass balance so that energy is conserved and the net flux is zero; (ii) flux bounds so that each flux (v) has a lower (*a*_n_) and upper (*b*_n_) flux rate, and others such as directionality and nutrient availability. This creates a constrained solution space that represents predictable solutions that are more biologically feasible. One example of CBM used in this illustration is flux balance analysis, where an objective function (*Z*, ATP utilisation) is defined and maximised for the optimal solution of *Z*, which can be identified within the solution space.

To address the diversity of data that has been accumulating for more than a decade, datasets have been tailored into genetically and biochemically consistent formats, mathematically structured ‘knowledge bases,’ such as GEMs (Papin et al., 2003; O’Brien et al., 2015). A GEM is an organised list of metabolic reactions derived from all available data of an organism’s metabolism. GEMs can be reconstructed into a mathematically structured network, a stoichiometric matrix *S* = (*m* × *n*) where *m* is the number of metabolites and *n* is the number of reactions to perform CBM, see Figure 2. It is often assumed that the system is at steady state, thus the net flux is null, *Sv* = 0, where v is the flux vector. Additional commonly used constraints are thermodynamic constraints to allow irreversibility of reactions, enzymatic capacity and availability of nutrients. These may also be applied so that GEMs can be analysed by CBM methods to target fluxes of metabolic reactions specific to phenotypic behaviour (e.g., growth or energy production) without the need for detailed kinetics, which are frequently not available. Initially, these models were used to represent single cell organisms with only relatively few metabolic reactions to maximise a desired phenotype, or an objective function, e.g., biomass (Price et al., 2003). However, metabolic reconstructions have since increased in size and scope, representing multicellular organisms with much larger genomes, such as Recon3D, the latest global reconstruction of human metabolism (Brunk et al., 2018). Some limitations to using GEMs for CBM methods is that arbitrary rates are typically used to model metabolic behaviour, and that classic CBM methods tend to lack the ability to model stochastic, complex behaviours, such as whole human cell metabolism. This can be due to a number of limiting factors of CBM, but one major controversy in the field is the use of a single defined objective function. Historically, this was used in biotechnology studies of single-cell organisms, where it was a simpler question to determine a single desired phenotype, e.g., growth or biomass. However, when modelling complex organisms where study of more than one phenotype is desirable, this becomes much less trivial. Although this continues to be debated, more recent advances in CBM have included the development of more sophisticated algorithms that allow modelling of more complex behaviours, as briefly discussed later in this review. Additionally, the majority of genome scale models are based on metabolism rather than signalling, although this area is also progressing (Hyduke and Palsson, 2010; Münzner et al., 2017).

#### Mitochondrial GEMs and CBMs

One predecessor of one of the first GEMs to study the functional mitochondrion using CBM was published in 2001 (Ramakrishna et al., 2001). This metabolic model included 46 metabolic reactions in two subcellular compartments, the mitochondrial matrix and cytosol with key shuttles, and included the glycolytic pathway, TCA cycle and OXPHOS. This model was simulated by a CBM method, termed flux balance analysis [FBA (Orth et al., 2010)]. In practise, FBA calculates a steady-state flux distribution, while maximising or minimising a desired reaction flux tagged as the “objective function.” This analysis gives an output of the optimal result for the objective function in question, within the biological solution space. As an initial cheque, FBA was used to predict energy metabolism (i.e., maximised ATP production) from the utilisation of various substrates (glucose, lactate, and palmitic acid) (Ramakrishna et al., 2001). The model predictions agreed with expected ATP yields from each substrate, and confirmed that glucose was the preferred energy substrate, determined by the maximum ATP production per mole of oxygen consumed. FBA was also performed independently to predict functional consequences of genetic knockouts (disabling individual metabolic reactions). Examples of genetic knockout simulations in the TCA cycle enzymes leading up to alpha-ketoglutarate, resulted in a lower rate of ATP production and an accumulation of oxaloacetate. Meanwhile, gene knockouts in the later stages of the TCA cycle from alpha-ketoglutarate dehydrogenase (mAKGD) to malate dehydrogenase also resulted in a lower rate of ATP production, but with an accumulation of alpha-ketoglutarate (Ramakrishna et al., 2001). This has encouraged confidence in the prediction results, since they correspond with the clinical observation of increased urinary excretion of alpha-ketoglutarate in patients with mAKGD, succinate dehydrogenase (SDH), and fumarase deficiencies (Rustin et al., 1997).

The first mitochondrial GEM was later generated by incorporating human cardiac mitochondrial proteomic and biochemical data, increasing the model to 189 reactions with 230 metabolites and 29 exchange reactions (Vo et al., 2004). In addition to the Ramakrishna model (Ramakrishna et al., 2001), pathways represented in the Vo model include fatty acid β-oxidation, phospholipid biosynthesis, urea cycle, and reactive oxygen species (ROS) detoxification. Initially, the biological solution space is created by constraints, including laws of thermodynamics to impose directionality, and can be further reduced with the addition of more constraints, e.g., enzymatic capacity based on experimental conditions or perturbations. Again, as a proof of principle study to build a GEM, CBM was used to characterise the model using FBA with three objective functions tested: ATP hydrolysis, phospholipid biosynthesis, and protohaem production (Vo et al., 2004). This study also utilised a different CBM, flux variability analysis (FVA) (Mahadevan and Schilling, 2003). FVA determines the maximum upper and lower flux bounds of all steady state reaction fluxes within the network while satisfying the optimal objective function, resulting in a solution space of flux distributions to determine what is physiologically feasible based on the condition of the model. This can reveal the level of model robustness in response to perturbations. The FVA simulation predicted that ATP production was the least flexible, while haem and phospholipid synthesis had greater flexibility (Vo et al., 2004). It is important to note that the less flexible a model response is, the less adaptable it is to maintain a steady state. The instability of metabolic reaction flux can then lead to the identification of critical points in dysregulation. With some modifications, the Vo model (Vo et al., 2004) was also utilised to investigate the impact of setting constraints that resembled diabetes, ischaemia, and low fat-high glucose (high carbohydrate) diet and high fat-low glucose (ketogenic) diet (Thiele et al., 2005). Network modifications included the addition of ketone body degradation and six transport reactions and the removal of 39 unused reactions, totalling 235 metabolites and 185 reactions, including 23 exchange reactions. Each simulated condition resulted in a reduction of network flexibility, rendering mitochondrial metabolism more sensitive to perturbations, such as changes in oxygen levels or higher ATP demands (Thiele et al., 2005). Although several therapies tested in this model were found to have only minimal effects, other therapies may be tested in future to identify potential targets which may have the greatest restoration of the network closest to the normal physiological condition.

Through the iterative cycles of GEM reconstruction, characterisation, testing and refinement, these sub-cellular models of metabolism contributed to the development of the first human whole cell metabolism models in 2007 (Duarte et al., 2007; Ma et al., 2007). These global models have since been utilised for various studies of human health and disease (Cook and Nielsen, 2017). As one of the first to use this methodology, Recon 1 (Duarte et al., 2007) was modified to represent the metabolism of human fibroblasts to study Leigh syndrome (Vo et al., 2007). This resulted in a network of 430 metabolites and 508 reactions, further expanding the previous mitochondrial model (Thiele et al., 2005) to include the pentose phosphate pathway, the malate-aspartate shuttle and *de novo* fatty acid synthesis. The study aimed to identify affected enzymes by non-invasively profiling the metabolic phenotype of normal and Leigh syndrome fibroblasts. This was performed by setting the model flux rates to isotopomer data of C^13^ labelled metabolites (pyruvate, lactate, glucose, and amino acids) from one control and one Leigh syndrome patient primary cell line and spent media. This study concluded that the Leigh syndrome-affected cell line had a slower metabolic rate and lower network flexibility within the respiratory chain enzyme fluxes. More specifically, succinate cytochrome *c* reductase (SCCR) enzyme activity was identified to be deficient with a higher activity ratio of cytochrome *c* oxidase/SCCR compared to control. Thus complex II deficiency was considered to be the most likely candidate for this particular Leigh syndrome-cell line. This provides an important step forward to alternative, non-invasive investigations to study the underlying mechanisms of a highly heterogeneous primary mitochondrial disease, such as Leigh syndrome. The interpretation of C^13^ isotopomer data analysis can then be enhanced by integrating it into a GEM for a more comprehensive coverage of mitochondrial metabolism. However, due to the lack of annotation, i.e., gene-protein reaction data, in Recon 1 at the time, further model improvements would be necessary in order to optimise such analyses. Another major limitation of this study was that the Leigh syndrome-affected cell line used in this work was not genetically characterised. Leigh syndrome is a heterogeneous disorder with more than 89 genetic causes described to date (Rahman et al., 2017). Thus it would be important to repeat these experiments in a series of Leigh syndrome cell cultures with different known gene defects, to determine whether there are common pathway abnormalities in all forms of Leigh syndrome or whether each gene defect has a unique profile. This would greatly impact biomarker discovery and therapy development.

A more comprehensive mitochondrial model was later generated, iAS253, a human heart mitochondrial model (Smith and Robinson, 2011). This model was manually reconstructed and annotated based on metabolite availability from the MitoMiner database (Smith and Robinson, 2009) and thoroughly annotated. iAS253 featured 253 reactions, 245 metabolites and 89 transport reactions. FBA was used to simulate perturbations, e.g., deficiency of fumarase, SDH and mAKGD, and to test dietary or supplementary therapeutic options *in silico*. This model showed high similarity between the *in silico* model features and clinical phenotypes, revealing possible disease mechanistic insights and initial stratification of potential therapeutic options. An expanded version of this model was later used to study OXPHOS disorders with deficiencies of complexes I–IV (Zielinski et al., 2016). Conclusions of this study were that complex I deficiency could be compensated by alternative pathways and complex II deficiency had lower metabolic flexibility leading to detrimental effects in both the TCA cycle and OXPHOS, whilst complexes III and IV deficiencies had the largest impact on ATP production. iAS253 has now been updated to MitoCore, which is currently the most comprehensive mitochondrial GEM to date (Smith et al., 2017). The model was manually upgraded to include 324 metabolic reactions, 83 transport steps between the mitochondrion and the cytosol, and 74 metabolite inputs and outputs through the plasma membrane. Initially, MitoCore was systematically compared with Recon 2.2, e.g., modelling fuel utilisation and fumarase deficiency, resulting in a more accurate representation of central metabolism (Smith et al., 2017).

More recently, MitoCore was used to investigate the effects of impaired mitochondrial citrate carrier (SLC25A1) function by FBA simulations (Majd et al., 2018). Whilst maintaining a minimal rate of ATP production, two objective functions were maximised; (i) fatty acid biosynthesis or (ii) glucose production via gluconeogenesis, with SLC25A1 specific reactions disabled to represent SLC25A1 deficiency. Although the majority of the connected pathways were sufficiently compensated by alternative pathways, the lack of citrate export had a detrimental effect on the production of acetyl-CoA required for fatty acid biosynthesis. The deficiency in acetyl-CoA would impair lipid, cholesterol, sphingolipid, and dolichol synthesis, all of which are vital for brain development, function and maintenance. In accordance with these predictions, these biosynthetic pathways also appear to be compromised in patients with missense mutations in *SLC25A1*, associated with an autosomal recessive neurometabolic disorder characterised by neonatal–onset encephalopathy. Future analyses could utilise this computational model to test therapeutic options for SLC25A1 deficiency.

MitoCore has also been used, after some modifications to include the production and efflux of tryptophan and lysine intermediates, to determine the effect of deficiency of the mitochondrial oxodicarboxylate carrier SLC25A21 on central metabolism (Boczonadi et al., 2018). The corresponding transport reactions of SLC25A21 were disabled during the FBA simulation while maximising ATP production. Simulation results showed that while ATP production and respiratory chain fluxes were maintained, there was an accumulation of lysine and tryptophan intermediates, L-pipecolic acid and quinolinic acid. The intermediate 2-oxoadipate was also found to be accumulated without affecting central metabolism in general. Furthermore, mass spectrometry analysis confirmed an accumulation of these three metabolites in patient urine samples compared to controls. Functional consequences were also tested *in vitro* by supplementing oxoadipate and quinolinic acid in control fibroblasts and neuronal SH-SY5Y cells, at equivalent concentrations to those measured in urine samples from affected patients using ultrahigh-performance liquid chromatography-tandem mass spectrometry. Although treated fibroblasts were largely unaffected, treated neuronal cells had decreased mitochondrial respiratory chain complexes and lower mtDNA copy number, resulting in induction of apoptosis. This implies that the intermediates are likely neurotoxic, which relates to the patient phenotype of a spinal muscular atrophy-like disease. Again, future analyses could use this model to identify critical points that could be targeted to allow restoration of normal levels of intermediates.

#### Other Systems Biological Models for Mitochondrial Research

An early GEM that represented the whole cell, rather than just the mitochondrion, included the Vo mitochondrial model (Vo et al., 2004; Duarte et al., 2007). Whole cell GEMs have evolved dramatically over the last decade, with the most recently published global GEM being Recon3D (Brunk et al., 2018). Recon3D can be accessed on the Virtual Metabolic Human [VMH, (Noronha et al., 2019)] and is graphically represented in ReconMap (Noronha et al., 2017). Several primary mitochondrial diseases have been mapped to Recon, including mitochondrial trifunctional protein deficiency, phosphate carrier deficiency and SUCLA2-related mtDNA depletion syndrome (Sahoo et al., 2012). Global GEM reconstructions have also been utilised for predictive modelling to represent multi-tissue systems (Bordbar et al., 2011). This could be a particularly useful approach to represent primary mitochondrial dysfunction in one or several tissues, and the interactions between tissues and their effects on the system as a whole. An example of a more detailed mitochondrion-centric model include a brain model with different cell types (Lewis et al., 2010). Such tissue-specific models could aid the eventual reconstruction of multi-tissue mitochondrial models.

Another common approach to computational modelling is the use of mathematical models, e.g., ordinary differential equations and kinetic data as parameters, to predict biological outcomes. There has also been a long historic use of mathematical models for mitochondrial basic science research, which is beyond the scope of this review. However, some noteworthy examples will be described briefly here. Many mathematical models have focused on modelling mitochondrial energy metabolism and signalling, calcium dynamics and regulation, ROS and redox, apoptosis, and fission and fusion. Historically, a popular mitochondrial model based on OXPHOS was made by Magnus and Keizer (1997) and other mitochondrial models were also developed independently by using different datasets and alternative biophysical theories, also focusing on OXPHOS (Korzeniewski, 1998, 2000; Korzeniewski and Zoladz, 2001; Beard, 2005). The Magnus and Keizer model (Magnus and Keizer, 1997) was widely used and adapted, e.g., to include metabolic pathways such as the TCA cycle, (Dudycha, 2000), calcium dynamics (Cortassa et al., 2003), and the production of ROS (Cortassa et al., 2004). Further model iterations for investigating these behaviours have since been developed, e.g., ROS (Gauthier et al., 2013), pH regulation and ion dynamics (Wei et al., 2011). Mitochondrial apoptosis has also been extensively investigated using this approach (Fussenegger et al., 2000; Rehm et al., 2006; Albeck et al., 2008; Bertaux et al., 2014). More recently, advances in computational and statistical approaches and the increasing availability of kinetic data have allowed these models to be developed to capture more mitochondrial behaviours, e.g., dynamic regulation of cellular metabolism and energetics (Dash et al., 2008; Zhang et al., 2018). Additionally, models have aimed to capture diversity within a mitochondrial population to investigate mitochondrial spatial and temporal dynamics (fission, fusion, mass, and motility) and heterogeneity by identifying sources of cell-to-cell variation of mitochondrial morphology and energetic stress states (Kowald and Kirkwood, 2011; Johnston et al., 2015; Dalmasso et al., 2017). Notably, kinetic modelling has covered a combination of metabolic and signalling models. It is a powerful method with its detailed use of reaction rates and metabolic concentrations for modelling, and can describe dynamic behaviour over time. However, this can become an incompatible method to model at a whole cell or tissue scale, since parameters may be limited or may not be suitable for the conditions in question.

A different type of predictive mitochondrial model recently built is Leigh Map (Rahman et al., 2017). Leigh Map was manually curated, incorporating >500 publications dated to November 2016 and information from the senior author’s clinical archive to include 89 genes and 237 phenotypes. This model is designed to be used as a diagnostic tool for Leigh syndrome to query a gene to identify associated phenotypes, or query phenotype(s) to identify the most likely causative gene(s). The efficiency of Leigh Map was found to be 80% identification of the correct gene in 20 Leigh syndrome cases (Rahman et al., 2017). Future work to expand the application of this tool to diagnose other primary mitochondrial diseases is ongoing.

## Challenges in the Systems Understanding of the Mitochondrion

Despite numerous advances, several challenges remain in the application of systems biology to mitochondrial research. The involvement of two genomes is an inherent challenge to the study of mitochondria. Varying mtDNA copy number and differing heteroplasmy levels between different cells and tissues are additional challenges (Guantes et al., 2016). These challenges are especially pronounced when studying the diversity of the multi-systemic phenotypes of primary mitochondrial diseases. Systems biology methods that can be used to address some of these issues include the use of whole cell GEMs, which have evolved to include the incorporation of both nuclear and mitochondrial genomes responsible for membrane transport, and have mapped several primary mitochondrial diseases (Sahoo et al., 2012). Additionally, whole-cell models can be applied to represent multiple tissues, and can be enhanced for mitochondrial research by the development of more detailed mitochondria-centric models (Lewis et al., 2010). In addition, other computational methods, e.g., agent-based modelling, have attempted to capture diversity within a mitochondrial population and cell-to-cell variation (Dalmasso et al., 2017). Mitochondrial disorders display significant phenotypic heterogeneity, even between individuals in the same family. This heterogeneity poses challenges for the diagnosis of mitochondrial diseases, which have begun to be addressed by a predictive diagnostic knowledgebase for Leigh syndrome (Rahman et al., 2017).

**FIGURE 3:**
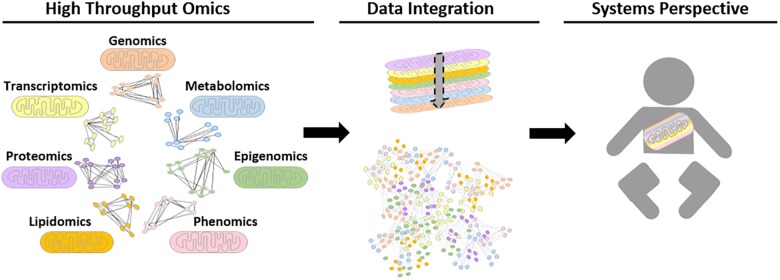
Schematic representation of possible future biological networks for mitochondrial systems biology. Different omics datasets can be generated from high throughput mitochondrial studies. To date, these are performed for single data types, for example using proteomic data to generate protein-protein interaction networks. However, future advances in computational tools could allow high-throughput omics data to be transformed into biological networks using mathematical algorithms, in order to find interactions and connections between biological moieties. Ultimately, the aim is to be able to integrate all relevant data types together to study the mitochondria within a whole system to improve patient care.

Analytical tools that could be used to understand mitochondrial biology and pathology using systems-level data are continuously evolving. Network-based approaches have played critical roles in the progression of systems biology, as they help illustrate complex molecular interactions. Until recently, a shortcoming of these networks was that network modalities were based on the analysis of a single datum type with manual comparisons to networks of different data types (Barabasi et al., 2011; Stevens et al., 2014; Wang et al., 2014). The impracticality of this approach led to the development of integrative network tools. There has also been a paradigm shift in the nature of data integration networks toward patient (population/study cohort)-centric approaches, such as SNF, which has not yet been explicitly used for mitochondrial datasets. Ultimately, the aim of this would be to develop more efficient integration tools to combine multi-omics datasets to gain further insight into mitochondrial biology and pathology, see Figure 3.

We have also discussed several examples of the use of CBM methods such as FBA and FVA which can provide predictive, valuable insights of the subject model. However, these results are only static snapshots of metabolism. Several methods have been developed to predict behaviour over time [dynamic FBA (Varma and Palsson, 1994) and dynamic FVA (Maldonado et al., 2018)], integrate regulation [regulatory FBA (Covert et al., 2001)] and integrate other data types [integrated FBA (Covert et al., 2008) and integrated dynamic FBA (Lee et al., 2008)]. Furthermore, other CBM methods and simulators have been developed to expand their applications, e.g., multi-objective function analyses (Costanza et al., 2012; Zakrzewski et al., 2012), whole human cell metabolic analyses (Fisher et al., 2013), integrate multiple simulation formats (Liao et al., 2012; Wu et al., 2016; Heirendt et al., 2017), of which all could be adapted for use in future primary mitochondrial research.

The generation and analysis of computational models have been shown to aid further insight into disease mechanisms and potential therapeutic discovery. However, the limitations of these network models are that they are only as accurate as the information input into them. Thus, these models have a tendency to only represent well-established pathways. However, as data and integration tools become increasingly available, there are well-characterised models such as MitoCore that could be built upon in the future. As we gain an increasing understanding of mitochondrial biology and pathophysiology, future computational models and accompanying analysis tools will be instrumental in improving diagnostic and therapeutic outcomes for primary mitochondrial disorders.

## Concluding Remarks

Systems biology research is still in early development for primary mitochondrial diseases. Since mitochondria are central organelles for many cellular functions across multiple tissues, the application of recent advances in systems biology will likely improve our understanding of mitochondrial diseases (Perocchi et al., 2006; Shutt and Shadel, 2007). The most recent predictive models MitoCore and Leigh Map provide useful examples of ongoing efforts using available mitochondrial resources, and the need to continue the iterative cycle of systems approaches to build, test and refine models for better computational representations. Indeed, as context-specific models become more comprehensive, global reconstructions will inevitably improve in future iterations and be more useful for mitochondrial research, made possible by utilising high throughput omics data (Palese and Bossis, 2012; Williams et al., 2016; Palmfeldt and Bross, 2017). For instance, GEMs may evolve to feature more detailed data and omics profiles specific to the subject of study, as these data become available (Argmann et al., 2016). This development will be of particular interest to represent the different types of primary mitochondrial diseases to characterise and identify what is common to all mitochondrial diseases and what is unique to particular gene defects or subgroups, for better diagnostic and therapeutic approaches.

Although current computational models have not yet reached standards needed for use in a clinical setting for diagnostic and therapeutic purposes, the quality of computational models has vastly improved in the last decade; the evolution of models is summarised in Table 2. Furthermore, computational modelling of mitochondrial biology can be enhanced by other datasets as described recently to gain mechanistic insight, progress therapeutic development and improve outcomes for patients with mitochondrial diseases (Rahman and Rahman, 2018). Systems biology will most certainly become more applicable to personalised medicine as these models evolve to encompass patient-tailored models, ultimately aiming for improved patient care.

**Table 2:** Model summary.

Model	Type of model	Model composition	Subject Studied	Reference
Ramakrishna	Mathematically structured mitochondrial-focussed metabolic model	46 reactions	ATP production; TCA enzyme deficiencies	Ramakrishna et al., 2001
Vo_1	Mitochondrial cardiomyocyte GEM	230 metabolites; 189 reactions	ATP production; haem synthesis; mixed phospholipid synthesis	Vo et al., 2004
Thiele	Mitochondrial GEM	235 metabolites; 185 reactions	Diabetes; Ischaemia; LF-HG and HF-LG diets	Thiele et al., 2005
Vo_2	Modified whole cell GEM	430 metabolites; 508 reactions	Leigh syndrome	Vo et al., 2007
MitoInteractome	PPIN: homology-based interaction modelling	6549 protein sequences across 74 species	Mitochondrial PPIN	Reja et al., 2009
Lewis	Modified whole cell GEMs to represent mitochondrial-centric multiple cell types	(i) 983 (ii) 983 (iii) 987 metabolites; (i) 1066 (ii) 1067 (iii) 1070 reactions	Alzheimer disease	Lewis et al., 2010
InterMitoBase	PPIN	5583 interactions between 2813 proteins	Mitochondrial PPIN	Gu et al., 2011
iAS253	Mitochondrial cardiomyocyte GEM	245 metabolites; 253 reactions	TCA enzyme deficiencies	Smith and Robinson, 2011
Mitochondrial protein functional (MPF) network	Based on network position, a scoring system for proteins in a PPIN ranging from 0–1, from most central to most peripheral	1254 mitochondrial proteins; 6071 functional links	Mitochondrial proteome from nine proteomic databases	Yang et al., 2013
Dalmasso	Mitochondrial-centric whole cell agent-based model	5 classes; 2 static and 3 dynamic agents	Cellular subpopulation mitochondrial dynamics	Dalmasso et al., 2017
Leigh map	Gene-to-phenotype network model	87 genes; 234 phenotypes	Leigh syndrome	Rahman et al., 2017
MitoCore	Mitochondrial cardiomyocyte GEM	441 metabolites; 491 reactions	ATP production; Proton leakage; Fumarase deficiency	Smith et al., 2017
xMWAS	Data integration and differential network analysis software	N/A	Mitochondrial Transcriptome-Metabolome-Wide Association Study	Go et al., 2018


*A descriptive outline of large scale models and tools described in this review.*

*Abbreviations: GEM, genome scale metabolic model; LF-HG, low fat-high glucose; HF-LG, high fat-low glucose; PPIN, protein–protein interaction network*.

## Author Contributions

EM, FT, JR, and SR contributed to conceptualisation, drafting, and critical revision of the manuscript.

## Conflict of Interest Statement

The authors declare that the research was conducted in the absence of any commercial or financial relationships that could be construed as a potential conflict of interest.
